# Supplementary material for: A Likelihood Approach for Real-Time Calibration of Stochastic Compartmental Epidemic Models
Source: PLoS Comput Biol. 2017 Jan 17;13(1):e1005257. doi: 10.1371/journal.pcbi.1005257 (PMC5240920; doi:10.1371/journal.pcbi.1005257)
Supplement: S1 File — (TAR.GZ) [file pcbi.1005257.s014.tar.gz › HSPH_Online-SI-Revision/EnKF07/n1-extreme/n1-extreme_EnKF_table0.pdf]

1	R0	{2.95019, 14.2854}	{2.50139, 4.88019}	{{2.13268, 0.604953}, {3., 8.60406}}	{16.8335, 66.3325}	inf	302.	54.1002	{8.78024, 104.198}	82.7958	0.977033	newly	137.	86.11	{12., 192.}	89.8905	0.667258	Reff	0.882107	0.599867	{0.122374, 1.51072}	65.9665	ar	924.	1782.1	{1605., 2437.}	98.855
2	R0	{2.67565, 16.946}	{2.63688, 6.21943}	{{1.74235, 2.72632}, {3., 11.4278}}	{17.1097, 64.1386}	inf	233.	40.4822	{4.82961, 79.9715}	85.4281	1.11326	newly	80.	42.56	{4., 109.}	87.85	0.752214	Reff	0.559211	0.564869	{0.107193, 1.19052}	58.4057	ar	926.	1765.93	{1615., 2152.}	90.7052
3	R0	{2.40066, 16.6543}	{2.35067, 5.50806}	{{0.521381, 1.61343}, {3., 10.201}}	{35.3708, 66.9271}	inf	237.	63.6071	{5.83091, 63.5947}	86.2453	0.929368	newly	91.	67.2	{5., 84.}	94.0879	0.614476	Reff	1.01788	0.441505	{0.0871355, 0.626483}	62.5985	ar	916.	1817.14	{1659., 1983.}	98.3777
4	R0	{1.9294, 15.4361}	{2.67654, 7.49078}	{{2.23243, 0.359277}, {3., 17.7072}}	{45.5935, 57.9559}	inf	135.	38.6525	{5.11551, 55.1527}	83.4693	0.852995	newly	58.	57.4	{4., 57.}	117.345	0.646258	Reff	1.07853	0.578718	{0.130402, 0.998386}	59.7402	ar	796.	1809.97	{1646., 2194.}	127.383
5	R0	{2.92859, 19.5393}	{2.5778, 3.33333}	{{2.01925, 0.0353775}, {3., 4.55001}}	{14.9043, 82.9404}	inf	261.	23.7309	{2.39263, 32.5727}	91.7613	1.3664	newly	98.	35.97	{2., 50.}	91.8265	0.894366	Reff	0.66479	0.406068	{0.0920068, 0.784811}	53.3598	ar	927.	1587.36	{1484., 1675.}	71.2362
6	R0	{1.79622, 12.3148}	{2.37957, 5.68392}	{{0.8938, 0.97043}, {3., 9.25114}}	{58.5076, 55.6988}	inf	97.	51.3151	{8.62731, 98.2138}	79.3416	0.611293	newly	44.	67.88	{8., 92.}	136.591	0.454825	Reff	0.941219	0.477578	{0.123156, 1.00168}	60.4153	ar	734.	1797.32	{1634., 2169.}	144.866
7	R0	{2.45855, 17.1776}	{2.35877, 6.90324}	{{1.63015, 2.90988}, {3., 12.9896}}	{24.9505, 59.8126}	inf	208.	69.1021	{7.01483, 124.099}	86.9086	0.884263	newly	81.	76.85	{5., 104.}	115.42	0.656736	Reff	0.98342	0.515349	{0.0959096, 1.01043}	61.9611	ar	871.	1901.76	{1670., 2493.}	118.342
8	R0	{2.20009, 15.519}	{2.64407, 6.27284}	{{1.96161, 3.21569}, {3., 8.312}}	{27.2478, 59.7147}	inf	171.	81.4335	{24.8525, 149.366}	76.3309	0.56786	newly	72.	81.03	{18., 160.}	98.0972	0.395197	Reff	0.708429	0.457063	{0.143339, 0.918727}	57.5869	ar	839.	1760.01	{1621., 1989.}	109.775
9	R0	{2.06493, 9.6414}	{2.48526, 5.52381}	{{2.00661, 0.984858}, {3., 9.85327}}	{30.6229, 45.0277}	inf	125.	43.4737	{9.30372, 63.886}	71.4588	0.683896	newly	96.	54.77	{6., 85.}	78.1146	0.643902	Reff	1.08409	0.476797	{0.0573553, 0.706988}	67.9009	ar	777.	1701.78	{1595., 2027.}	122.543
10	R0	{2.60775, 19.6936}	{2.63869, 3.69652}	{{1.93981, 2.01699}, {3., 7.61921}}	{18.0952, 81.2298}	inf	215.	27.7702	{3.35743, 42.6564}	87.5045	1.12861	newly	62.	59.13	{4., 54.}	108.984	0.615927	Reff	0.904889	0.484488	{0.0823444, 0.673422}	59.5802	ar	889.	1630.7	{1517., 1678.}	83.4308
11	R0	{1.99168, 8.13555}	{2.34264, 6.99431}	{{1.66317, 2.88305}, {3., 13.6568}}	{31.527, 48.4027}	inf	102.	68.0149	{5.64531, 213.52}	95.3729	0.703164	newly	81.	88.32	{5., 109.}	136.716	0.730237	Reff	0.938081	0.528238	{0.0960959, 1.66033}	63.3266	ar	772.	1901.23	{1653., 2393.}	146.273
12	R0	{1.79304, 9.18941}	{2.44961, 5.40682}	{{1.19343, 0.596875}, {3., 9.61389}}	{46.5923, 45.6301}	inf	134.	54.8864	{13.2173, 102.23}	73.5604	0.642932	newly	109.	74.94	{13., 156.}	88.2202	0.603426	Reff	1.0059	0.595729	{0.150872, 1.56481}	63.333	ar	725.	1813.58	{1631., 2365.}	152.223
13	R0	{1.84016, 18.3431}	{2.57601, 5.3235}	{{1.93494, 0.383786}, {3., 13.2911}}	{39.9882, 70.9782}	inf	100.	69.9926	{8.6857, 211.796}	97.4159	0.638449	newly	41.	75.7	{7., 188.}	151.659	0.398482	Reff	1.13354	0.467471	{0.13094, 0.774305}	67.2838	ar	762.	1785.75	{1597., 2276.}	134.35
14	R0	{1.94221, 9.85971}	{2.59921, 6.63654}	{{2.2423, 3.81074}, {3., 15.6403}}	{42.8614, 51.1011}	inf	139.	37.7531	{7.66567, 63.7925}	79.4876	0.787117	newly	92.	45.52	{7., 64.}	85.9348	0.72341	Reff	0.594316	0.47864	{0.113406, 0.749788}	43.9516	ar	805.	1734.39	{1630., 1860.}	115.452
15	R0	{1.78578, 12.0541}	{2.37394, 4.83869}	{{1.11812, 2.64974}, {3., 12.9095}}	{55.7741, 61.6782}	inf	118.	50.0208	{6.74527, 70.4207}	90.5355	0.796194	newly	65.	52.24	{6., 65.}	97.6923	0.595379	Reff	0.878604	0.443565	{0.168482, 0.653115}	55.9521	ar	732.	1686.06	{1532., 1836.}	130.336
16	R0	{1.94463, 15.6503}	{2.40045, 6.0849}	{{1.63682, 3.67972}, {3., 9.28077}}	{34.2653, 61.1196}	inf	143.	67.0902	{3.77695, 124.614}	92.9043	0.838139	newly	51.	81.69	{4., 247.}	162.059	0.587837	Reff	0.958703	0.512237	{0.0544289, 1.38638}	63.2133	ar	757.	1875.89	{1640., 2459.}	147.806
17	R0	{2.08901, 16.4314}	{2.65028, 7.25176}	{{1.78717, 2.50001}, {3., 17.4068}}	{34.2153, 59.0019}	inf	160.	54.8413	{8.14927, 138.128}	83.2565	0.823017	newly	61.	63.8	{6., 129.}	120.852	0.58747	Reff	1.04868	0.566461	{0.0930346, 1.23346}	59.4107	ar	826.	1789.3	{1603., 2203.}	116.622
18	R0	{2.76419, 18.56}	{2.32044, 6.86953}	{{1.64709, 3.33305}, {3., 13.0851}}	{23.7312, 62.9874}	inf	275.	79.9434	{7.42029, 126.915}	87.5766	0.96588	newly	103.	78.67	{7., 124.}	111.	0.726731	Reff	0.931532	0.444694	{0.0555034, 0.78399}	65.4034	ar	920.	1858.78	{1650., 2439.}	102.041
19	R0	{2.3821, 17.1399}	{2.47461, 6.49926}	{{1.19309, 1.44227}, {3., 10.196}}	{23.3651, 62.0811}	inf	229.	61.2362	{8.97128, 125.129}	81.3846	0.834734	newly	77.	72.06	{8., 124.}	99.3506	0.542214	Reff	1.07433	0.534253	{0.0907253, 1.19146}	66.8359	ar	883.	1734.8	{1585., 2299.}	99.2707
20	R0	{2.78314, 19.6127}	{2.66569, 5.46059}	{{1.92322, 1.4635}, {3., 7.91897}}	{14.8171, 72.1579}	inf	236.	40.9002	{5.5964, 61.0189}	85.9455	0.990533	newly	84.	47.17	{5., 59.}	83.5357	0.643685	Reff	0.411905	0.469376	{0.177376, 0.761629}	45.9077	ar	943.	1649.38	{1552., 1724.}	74.9077
21	R0	{1.85124, 14.6794}	{2.59375, 6.65469}	{{1.80003, 1.08077}, {3., 12.6295}}	{43.3191, 56.7894}	inf	97.	57.023	{3.04145, 129.083}	102.252	0.830445	newly	43.	62.19	{2., 201.}	164.442	0.703195	Reff	1.20516	0.45839	{0.0786856, 0.768003}	67.4918	ar	730.	1795.58	{1618., 2403.}	145.97
22	R0	{2.1028, 14.9852}	{2.70503, 6.68096}	{{2.24948, 1.18919}, {3., 15.2349}}	{33.0948, 58.177}	inf	167.	38.65	{4.77451, 68.5005}	82.4191	0.907961	newly	63.	73.87	{5., 54.}	138.524	0.650215	Reff	0.738083	0.582713	{0.147912, 0.878638}	55.3799	ar	840.	1812.35	{1644., 2173.}	115.756
23	R0	{1.86601, 10.2157}	{2.36344, 6.43582}	{{1.64156, 3.40619}, {3., 11.8336}}	{36.7618, 45.2413}	inf	118.	69.2486	{5.77656, 146.241}	94.8294	0.729014	newly	79.	76.72	{6., 106.}	121.975	0.680622	Reff	1.02071	0.492035	{0.0926133, 0.808785}	64.0889	ar	747.	1870.47	{1643., 2397.}	150.398
24	R0	{2.34032, 10.0992}	{2.47054, 4.81692}	{{1.16353, 0.441914}, {3., 7.42746}}	{24.7365, 53.324}	inf	202.	65.5052	{10.8124, 112.108}	80.885	0.754393	newly	137.	111.83	{12., 272.}	104.781	0.641248	Reff	0.840175	0.5942	{0.0946093, 1.45877}	64.6142	ar	887.	1802.94	{1621., 2510.}	108.812
25	R0	{1.91548, 15.3401}	{2.6482, 8.48641}	{{2.22756, 3.82651}, {3., 16.3744}}	{47.566, 48.8743}	inf	146.	33.93	{5.76714, 48.0865}	79.8879	0.788896	newly	68.	31.05	{6., 42.}	80.4853	0.62437	Reff	1.10523	0.451443	{0.0784622, 0.770266}	64.2275	ar	765.	1739.19	{1642., 1892.}	127.345
26	R0	{2.75283, 10.6721}	{2.53837, 6.23649}	{{1.63455, 3.60002}, {3., 15.0806}}	{18.3853, 52.8314}	inf	255.	82.7553	{4.52345, 273.522}	86.3681	0.987885	newly	153.	97.86	{6., 392.}	110.078	0.914844	Reff	0.743264	0.498763	{0.114848, 0.64233}	53.0359	ar	926.	1864.44	{1646., 2374.}	101.343
27	R0	{2.08367, 19.7849}	{2.8273, 7.86579}	{{2.42881, 0.583401}, {3., 14.7503}}	{35.6883, 60.2435}	inf	158.	52.993	{6.31279, 64.1657}	87.9872	0.851233	newly	54.	49.66	{4., 54.}	112.926	0.613103	Reff	0.927233	0.514691	{0.126075, 0.942323}	59.9867	ar	833.	1787.01	{1613., 2215.}	114.527
28	R0	{2.64334, 17.1765}	{2.39261, 8.62152}	{{0.608539, 0.595833}, {3., 18.1557}}	{27.8354, 51.9312}	inf	230.	73.665	{8.21511, 166.426}	82.8561	0.824168	newly	99.	74.08	{8., 84.}	101.919	0.675649	Reff	0.734849	0.437434	{0.0938183, 0.636327}	50.1026	ar	934.	1822.5	{1644., 2113.}	95.1285
29	R0	{2.87008, 13.3872}	{2.5144, 5.23919}	{{0.744006, 0.673748}, {3., 10.7634}}	{18.5525, 62.0681}	inf	267.	73.1378	{5.04152, 170.871}	83.0264	0.96687	newly	113.	115.22	{4., 459.}	123.522	0.754041	Reff	0.608457	0.623144	{0.120932, 1.43896}	60.0069	ar	941.	1874.97	{1617., 2442.}	99.2529
30	R0	{2.06105, 19.6275}	{2.63498, 6.21748}	{{2.12252, 0.465018}, {3., 12.7534}}	{28.9204, 68.3522}	inf	100.	77.7904	{8.62119, 83.6273}	90.4521	0.533053	newly	41.	101.08	{7., 104.}	206.293	0.394519	Reff	0.715184	0.563579	{0.147892, 1.24137}	62.908	ar	747.	1763.37	{1537., 2212.}	136.06
31	R0	{2.30764, 13.435}	{2.39454, 6.61641}	{{1.62788, 3.71206}, {3., 13.6385}}	{27.1521, 51.6931}	inf	231.	81.4518	{5.46745, 274.596}	86.2335	0.94528	newly	94.	88.74	{4., 151.}	120.	0.740487	Reff	0.717676	0.497901	{0.0902733, 0.911105}	56.1948	ar	877.	1924.47	{1678., 2555.}	119.438
32	R0	{2.30565, 11.9237}	{2.37861, 7.24168}	{{1.63165, 1.59556}, {3., 13.3696}}	{27.1831, 44.5261}	inf	219.	91.715	{3.51589, 297.523}	88.3177	0.943091	newly	128.	88.39	{6., 216.}	103.727	0.85921	Reff	0.788532	0.50886	{0.0851476, 0.897329}	56.8803	ar	853.	1922.36	{1672., 2625.}	125.508
33	R0	{2.24031, 18.582}	{2.32385, 4.76128}	{{0.812441, 0.105564}, {3., 9.29502}}	{38.8395, 74.6026}	inf	205.	49.1011	{8.41028, 115.076}	80.4679	0.866349	newly	78.	98.27	{8., 310.}	135.397	0.611735	Reff	1.10895	0.558137	{0.111162, 0.933152}	61.4443	ar	864.	1777.66	{1600., 2157.}	105.748
34	R0	{2.33306, 15.9759}	{2.33381, 7.266}	{{0.643529, 0.58842}, {3., 18.1673}}	{37.4891, 59.1616}	inf	204.	70.4669	{9.99248, 124.015}	85.558	0.821077	newly	96.	74.13	{9., 81.}	103.927	0.650941	Reff	0.874898	0.412935	{0.0841421, 0.642885}	56.3559	ar	854.	1794.95	{1628., 1975.}	110.181
35	R0	{2.48357, 18.3573}	{2.36335, 6.38581}	{{1.59941, 3.86542}, {3., 12.1566}}	{23.6604, 65.2138}	inf	218.	67.2241	{3.61972, 96.3054}	88.5801	0.987632	newly	79.	88.38	{3., 103.}	139.241	0.738687	Reff	1.0282	0.515996	{0.07334, 1.51014}	63.7736	ar	835.	1886.56	{1650., 2488.}	125.935
36	R0	{2.59548, 19.9046}	{2.68246, 6.2725}	{{1.80593, 3.24096}, {3., 11.5003}}	{17.2331, 68.4872}	inf	240.	38.7767	{10.882, 65.797}	85.5313	1.00391	newly	83.	44.07	{10., 82.}	83.8675	0.621205	Reff	0.931777	0.544314	{0.0716688, 1.20377}	57.4218	ar	863.	1752.98	{1639., 1934.}	103.126
37	R0	{2.29641, 16.9868}	{2.78861, 5.37104}	{{2.44757, 0.702906}, {3., 18.6422}}	{24.5661, 70.7331}	inf	186.	36.0819	{2.66451, 62.5226}	86.5131	1.11402	newly	65.	47.22	{3., 106.}	106.246	0.748979	Reff	0.803743	0.518613	{0.269891, 0.791111}	46.787	ar	877.	1652.19	{1511., 1980.}	88.3911
38	R0	{2.65743, 15.4569}	{2.70055, 6.60445}	{{1.78534, 3.43219}, {3., 13.5993}}	{15.8445, 59.9422}	inf	233.	41.0847	{4.06737, 76.467}	85.0499	1.0575	newly	114.	43.03	{3., 91.}	83.3421	0.85082	Reff	0.733451	0.5381	{0.0913803, 1.06168}	51.8605	ar	897.	1772.63	{1630., 2006.}	97.6176
39	R0	{2.67953, 14.5132}	{2.40397, 6.51341}	{{1.62457, 3.89715}, {3., 14.2648}}	{21.6567, 56.5255}	inf	233.	73.4075	{5.32399, 168.287}	90.318	1.00273	newly	117.	74.93	{5., 98.}	108.128	0.830591	Reff	1.15488	0.514795	{0.0625341, 0.796775}	64.4419	ar	894.	1872.38	{1654., 2388.}	109.438
40	R0	{1.94594, 11.928}	{2.67833, 6.11032}	{{2.25852, 0.92934}, {3., 16.5395}}	{44.6829, 64.5743}	inf	115.	48.7628	{4.6081, 62.154}	86.9867	0.74984	newly	66.	67.08	{6., 79.}	118.364	0.575831	Reff	0.8387	0.515263	{0.154272, 0.782987}	58.4694	ar	796.	1809.69	{1650., 2224.}	127.348
41	R0	{1.66297, 10.0047}	{2.58004, 5.15708}	{{1.19225, 2.86489}, {3., 12.4841}}	{67.4576, 55.5423}	inf	73.	47.1762	{5.34752, 58.8327}	94.8183	0.633047	newly	55.	45.35	{4., 51.}	99.1455	0.564346	Reff	0.964523	0.4369	{0.145825, 0.715639}	59.2174	ar	716.	1679.57	{1558., 1781.}	134.577
42	R0	{2.91568, 16.4453}	{2.45329, 5.58522}	{{1.24857, 1.00258}, {3., 9.73527}}	{18.6929, 66.2614}	inf	275.	61.9426	{11.034, 145.26}	80.3952	0.881602	newly	105.	73.99	{14., 159.}	83.0952	0.546628	Reff	0.906776	0.638791	{0.150387, 1.71878}	61.5411	ar	919.	1812.25	{1585., 2555.}	99.8683
43	R0	{2.55898, 15.8889}	{2.36193, 6.19013}	{{1.64556, 3.56251}, {3., 9.27147}}	{23.211, 61.1856}	inf	219.	78.5271	{4.91926, 277.724}	90.3837	0.973409	newly	75.	98.58	{4., 388.}	155.093	0.703657	Reff	1.07989	0.514209	{0.0713016, 1.58954}	64.8399	ar	904.	1891.18	{1639., 2450.}	109.201
44	R0	{2.28649, 15.9017}	{2.69989, 7.03265}	{{2.22199, 1.07829}, {3., 15.3008}}	{23.7922, 56.7777}	inf	219.	32.178	{2.92302, 60.5782}	85.3427	1.07038	newly	76.	48.61	{4., 58.}	99.7237	0.747125	Reff	0.756828	0.537889	{0.156324, 0.918019}	50.7469	ar	880.	1772.17	{1639., 2067.}	101.383
45	R0	{2.65121, 18.6339}	{2.4484, 5.52708}	{{1.07513, 0.596468}, {3., 9.55451}}	{20.9065, 70.3386}	inf	260.	56.0947	{14.2449, 123.729}	79.3956	0.856565	newly	105.	75.12	{11., 140.}	86.9524	0.584371	Reff	1.01011	0.552527	{0.151696, 1.22836}	64.3556	ar	928.	1769.6	{1608., 2239.}	94.3147
46	R0	{2.52291, 18.0251}	{2.56646, 6.49178}	{{2.21472, 0.455338}, {3., 15.8225}}	{20.057, 64.5072}	inf	228.	34.0833	{6.46861, 55.6673}	86.0757	1.02515	newly	79.	57.28	{4., 64.}	103.038	0.696514	Reff	0.915816	0.543752	{0.138837, 0.933345}	55.4811	ar	894.	1792.82	{1644., 2103.}	100.539
47	R0	{2.22618, 17.1225}	{2.24015, 5.381}	{{0.598616, 1.11497}, {3., 11.5107}}	{42.8613, 68.5735}	inf	176.	63.2661	{8.33793, 107.378}	86.4929	0.826155	newly	67.	72.16	{6., 79.}	107.731	0.527445	Reff	1.15316	0.413708	{0.0935512, 0.694215}	68.8396	ar	850.	1828.79	{1670., 1988.}	115.152
48	R0	{2.04405, 16.073}	{2.26759, 8.60869}	{{0.631302, 0.583166}, {3., 18.0305}}	{49.4059, 52.049}	inf	137.	68.9903	{5.23922, 150.626}	88.376	0.736771	newly	68.	68.32	{4., 85.}	115.794	0.633256	Reff	1.11196	0.418946	{0.0684255, 0.651379}	67.8395	ar	779.	1798.46	{1637., 2042.}	131.343
49	R0	{2.84396, 18.097}	{2.4382, 6.47437}	{{1.16609, 1.27953}, {3., 12.6521}}	{19.5348, 64.2241}	inf	289.	75.8913	{13.8052, 172.732}	76.2271	0.837984	newly	115.	84.46	{12., 259.}	85.7217	0.595259	Reff	0.665487	0.547317	{0.0430214, 1.38301}	68.7556	ar	952.	1844.39	{1633., 2540.}	96.2826
50	R0	{1.85341, 18.0975}	{2.27023, 7.65165}	{{0.7111, 2.86121}, {3., 18.1856}}	{56.998, 58.6733}	inf	148.	60.3797	{6.9185, 105.621}	84.0612	0.764054	newly	54.	51.09	{4., 70.}	96.8704	0.539543	Reff	0.850715	0.373255	{0.117286, 0.557899}	60.0271	ar	754.	1796.09	{1638., 2067.}	138.208
